# Risks of Dementia in a General Japanese Older Population With Preserved Ratio Impaired Spirometry: The Hisayama Study

**DOI:** 10.2188/jea.JE20230207

**Published:** 2024-07-05

**Authors:** Kenji Kawatoko, Yasuyoshi Washio, Tomoyuki Ohara, Satoru Fukuyama, Takanori Honda, Jun Hata, Taro Nakazawa, Keiko Kan-o, Hiromasa Inoue, Koichiro Matsumoto, Tomohiro Nakao, Takanari Kitazono, Isamu Okamoto, Toshiharu Ninomiya

**Affiliations:** 1 Department of Respiratory Medicine, Graduate School of Medical Sciences, Kyushu University, Fukuoka, Japan; 2 Department of Epidemiology and Public Health, Graduate School of Medical Sciences, Kyushu University, Fukuoka, Japan; 3 Department of Neuropsychiatry, Graduate School of Medical Sciences, Kyushu University, Fukuoka, Japan; 4 Department of Respiratory Medicine, National Hospital Organization Omuta National Hospital, Fukuoka, Japan; 5 Center for Cohort Studies, Graduate School of Medical Sciences, Kyushu University, Fukuoka, Japan; 6 Department of Medicine and Clinical Science, Graduate School of Medical Sciences, Kyushu University, Fukuoka, Japan; 7 Department of Pulmonary Medicine, Graduate School of Medical and Dental Sciences, Kagoshima University, Kagoshima, Japan; 8 Division of Respiratory Medicine, Fukuoka Dental College Medical and Dental Hospital, Fukuoka, Japan

**Keywords:** preserved ratio impaired spirometry, spirometry classification, dementia, prospective cohort study

## Abstract

**Background:**

Studies on the association between preserved ratio impaired spirometry (PRISm) and dementia are limited. Indeed, PRISm has often been overlooked or ignored as an index of lung function impairment. Therefore, we investigated the association of PRISm with the risk for the development of dementia in an older Japanese population.

**Methods:**

A total of 1,202 community-dwelling, older Japanese participants aged ≥65 years without dementia were followed up for a median of 5.0 years. Participants were categorized by spirometry as follows: normal spirometry (FEV_1_/FVC ≥0.70 and FEV_1_ ≥80% predicted), PRISm (≥0.70 and <80%), airflow limitation (AFL) Global Initiative for Chronic Obstructive Lung Disease (GOLD) 1 (<0.70 and ≥80%), and AFL GOLD 2 to 4 (<0.70 and <80%). Hazard ratios (HRs) and their 95% confidence intervals (CIs) were computed using a Cox proportional hazards model.

**Results:**

During the follow-up period, 122 participants developed dementia. The age- and sex-adjusted incidences of dementia in the participants with normal spirometry, PRISm, AFL GOLD 1, and AFL GOLD 2 to 4 were 20.5, 37.0, 18.4, and 28.6 per 1,000 person-years, respectively. Participants with PRISm had a higher risk of dementia (HR 2.04; 95% CI, 1.19–3.49) than those with normal spirometry after adjusting for confounders. Moreover, both reduced FEV_1_% predicted values and FVC% predicted values were associated with the risk of dementia.

**Conclusion:**

PRISm was associated with an increased risk of dementia in a general older Japanese population.

## INTRODUCTION

Dementia is considered a major public health problem because of its psychological, social, and economic impacts.^1^ The importance of early risk assessment and reduction for dementia has increased along with the increasing number of people living with dementia worldwide.^2^ Several modifiable factors, such as diabetes, obesity, smoking, depression, and hypertension, are known risk factors for dementia.^1^^,^^3^ Previous population-based studies have also suggested that chronic lung diseases and lung function decline, such as lung function impairment, airflow limitation (AFL), and restrictive lung function, were potential risk factors for dementia or cognitive impairment.^4^^–^^10^

Recently, preserved ratio impaired spirometry (PRISm), defined as preserved forced expiratory volume in 1 second (FEV_1_)/forced vital capacity (FVC) ratio but reduced FEV_1_,^11^^,^^12^ has been assessed as a subtype of lung function impairment assessed by spirometry, and has played an increasingly important role in clinical and public health practices. Several population-based studies have reported that individuals with PRISm exhibit increased respiratory symptoms, development of AFL, cardiovascular outcomes, and mortality.^12^^–^^18^ Although its prevalence in the general population has been reported to be between 7.1% and 20.3%,^12^^–^^20^ PRISm has often been ignored or overlooked in clinical studies. It was generally recognized as being a Global Initiative for Chronic Obstructive Lung Disease (GOLD)-unclassified, nonspecific, or normal spirometry pattern because its FEV_1_/FVC is preserved.^21^^,^^22^ In addition, population-based studies on the association between PRISm and dementia have been limited. Indeed, there has been only one population-based study in a Western population—which found that participants with PRISm had an elevated risk for developing dementia^4^—and there have been no studies examining the association between PRISm and the development of dementia in other populations, including East Asians. Considering the racial and ethnic differences in each of the spirometric reference indices, including FEV_1_, FVC, and FEV_1_/FVC, and the incidence of dementia,^23^^–^^25^ investigation of the association between PRISm and developing dementia in a general population—and particularly a general East Asian population, since collectively the East Asian countries comprise one of the largest populations in the world^26^—is important. The present study aimed to clarify the association between PRISm and the risks for the development of dementia in an older Japanese population.

## METHODS

### Study population and data

We conducted a prospective study using the data from the Hisayama Study. The Hisayama Study is an ongoing population-based longitudinal study of cerebro-cardiovascular diseases conducted since 1961 in Hisayama Town, a suburb of the Fukuoka metropolitan area on Kyushu Island, Japan; comprehensive surveys of cognitive impairment in the older adult population have been conducted since 1985.^27^ A detailed description of this survey has been published previously.^28^ We used the baseline data obtained between 2012 and 2013. Briefly, a total of 1,906 residents in Hisayama town aged ≥65 years (participation rate: 93.6%) participated in the cognitive function survey from 2012 to 2013. In addition, a comprehensive health examination including spirometry and risk factor assessments was performed from 2012 to 2013. After excluding 44 participants who did not consent to participate in the epidemiologic studies, 2 participants without available data on cognitive function, 2 participants with disturbance of consciousness or mental retardation, 339 participants with dementia at baseline, 163 participants who did not participate in the comprehensive health examination, and 154 participants without available spirometry data, the remaining 1,202 participants (669 women and 533 men) were enrolled in the present study (eFigure 1). This study was approved by the Kyushu University Institutional Review Board for Clinical Research (approval no. 2023-56), and written informed consent was obtained from all participants.

### Definition of PRISm and AFL

Lung function examination using spirometry was performed at the baseline examination according to the guidelines of the Japanese Respiratory Society.^29^ To obtain adequate flow-volume loops without bronchodilators, we performed at least two and up to four measurements using a CHESTGRAPH HI-105 electronic spirometer (ChestMI, Tokyo, Japan). Pulmonologists visually assessed the quality of the maneuvers and selected the best flow-volume loop, defined as the loop with the highest sum of FEV_1_ and FVC. The predicted FEV_1_ and FVC were calculated using the reference equations for the Japanese population as reported by the Clinical Pulmonary Functions Committee of the Japanese Respiratory Society in 2014.^30^ Participants were classified into the following spirometry categories according to the GOLD criteria and previous reports^11^^,^^15^^,^^16^: normal spirometry (FEV_1_/FVC ≥0.70 and FEV_1_ ≥80% predicted), PRISm (FEV_1_/FVC ≥0.70 and FEV_1_ <80% predicted), AFL GOLD 1 (FEV_1_/FVC <0.70 and FEV_1_ ≥80% predicted), and AFL GOLD 2 to 4 (FEV_1_/FVC <0.70 and FEV_1_ <80% predicted).

### Diagnosis of dementia

We conducted comprehensive cognitive screening examinations, including neuropsychological tests, such as the Mini-Mental State Examination,^31^ over a baseline period from 2012 to 2013 and a follow-up period from 2017 to 2018. If a participant was suspected of having neurological symptoms, including cognitive impairment, a comprehensive evaluation was conducted by an expert psychiatrist.^28^ Dementia and mild cognitive impairment (MCI) were diagnosed according to the guidelines of the Diagnostic and Statistical Manual of Mental Disorders, Third Edition, Revised,^32^ and the clinical criteria reported by Petersen et al,^33^ respectively. MCI was defined as the presence of either of (1) objective cognitive impairment based on the neuropsychological data or (2) any cognitive complaint by a family member, municipal health staff, welfare office staff, or local practitioner in participants who showed no evidence of dementia. Expert psychiatrists and stroke physicians discussed the diagnosis of dementia for each case of dementia and MCI.

### Follow-up survey

Participants were followed for a median of 5.0 years (interquartile range: 4.8 to 5.1 years) from the baseline survey to the date of the follow-up neuropsychological survey for dementia in 2017 to 2018. Those who did not participate in the follow-up survey were followed up until March 31, 2018. Except for deceased cases, no participants were lost to follow-up. Detailed methods of the dementia follow-up survey have been reported previously.^28^ To collect information on new neuropsychiatric events including dementia and stroke, we established a daily monitoring system among the study group, local physicians, and municipal health and welfare office staff. In this monitoring system, the physicians in the study group collect information on dementia and stroke events, including suspected cases, by regularly visiting clinics, hospitals, and the town’s office. We also collected information on new cases of dementia and stroke at annual health examinations. Health information was checked by letter or telephone for participants who did not have an annual health examination or who moved out of the town. Participants with suspected dementia or any neurological symptoms were carefully evaluated by a psychiatrist and a stroke physician on the study team. In addition, when a participant died, we collected and reviewed all available information, including neuroimaging and interviews with the family or treating physician.

### Clinical assessments and laboratory measurements

At the baseline examination, each participant completed a self-administered questionnaire on educational status, smoking habits, cumulative cigarettes as pack-years, alcohol intake, regular exercise, treatment with antihypertensive agents, glucose-lowering agents, lipid-modifying agents, bronchodilators, and inhaled corticosteroids (ICSs). The questionnaire was administered by trained interviewers. Total energy intakes were estimated using a semi-quantitative food frequency questionnaire.^34^ Education level was categorized as either ≤9 or >9 years of formal education. Smoking habits were categorized as never, former, and current smoker. Pack-years of smoking were calculated as cigarettes per day multiplied by years of smoking divided by 20. Alcohol intake was classified as current habitual drinker or not. Regular exercise was defined as participating in sports or other leisure-time exercise activity at least three times per week. Bronchodilators were defined as short- or long-acting β_2_ agonists or muscarinic antagonists, or xanthines. ICSs were defined as any ICS, such as fluticasone propionate, mometasone furoate, ciclesonide, or budesonide. Height and weight were measured in light clothing without shoes, and body mass index (BMI) was calculated (kilograms per meter squared). Blood pressure was measured three times with an automated sphygmomanometer after resting for at least 5 minutes in the sitting position. The mean of the three measurements was used in the analyses. Hypertension was defined as systolic blood pressure ≥140 mm Hg, diastolic blood pressure ≥90 mm Hg, or current treatment with antihypertensive agents. Diabetes mellitus was defined as fasting plasma glucose ≥7.0 mmol/L, 2-h post load or casual glucose ≥11.1 mmol/L, or treatment with current oral antidiabetic agents or insulin. Serum total and high-density lipoprotein (HDL) cholesterol levels were measured enzymatically, and dyslipidemia was defined as serum total cholesterol ≥5.7 mmol/L, serum HDL cholesterol <1.03 mmol/L, or current treatment with lipid-modifying agents. Serum creatinine concentrations were measured using an enzymatic method, and the estimated glomerular filtration rate (eGFR) was calculated using the Japanese coefficient-modified Chronic Kidney Disease Epidemiology Collaboration equation.^35^ Kidney dysfunction was defined as eGFR <45 mL/min/1.73 m^2^. Handgrip strength was measured using a digital strength dynamometer (T.K.K.5401; Takei Scientific Instruments, Niigata, Japan), as instructed by trained personnel or a nurse. The participants were encouraged to exert maximal handgrip strength. Two trials were recorded alternately for each hand, and the maximum of four measurements was used. Serum N-terminal pro-B-type natriuretic peptide (NT-proBNP) levels were measured using an Elecsys proBNP Immunoassay (Roche Diagnostics, Rotkreuz, Switzerland). History of stroke was defined as any previous cerebrovascular event of symptomatic stroke, including ischemic stroke, intracerebral hemorrhage, and subarachnoid hemorrhage as determined by all available clinical information and medical records.

### Statistical analysis

For each of the four lung function categories, means of continuous variables and frequencies of categorical variables were compared using analysis of variance with Dunnett’s test for multiple comparisons and logistic regression analysis with normal spirometry as the reference. For baseline characteristics of the population, pack-years and serum NT-proBNP levels were reported as the median and interquartile range. The NT-proBNP level was log-transformed in the analysis to an approximately normal distribution before statistical tests were performed. Pack-years were compared using the Wilcoxon rank-sum test for each lung function category. The incidence of dementia was calculated using the person-year method with adjustment for age and sex. Hazard ratios (HRs) with 95% confidence intervals (CIs) for the development of dementia were estimated using Cox proportional hazards models. We performed multivariable-adjusted analysis, and adjusted the risk estimates for potential confounders at baseline as follows: model 1, adjusted for age and sex; model 2, adjusted for age, sex, education level, body mass index, systolic blood pressure, use of antihypertensive agents, serum total cholesterol, serum HDL cholesterol, lipid-modifying agents, diabetes, history of stroke, current smoking, pack-years, current drinking, and regular exercise. The heterogeneity in the association of lung function category with the risk of dementia between subgroups was assessed by adding multiplicative interaction terms to the respective Cox model. For this particular analysis we excluded 301 participants with AFL (GOLD 1 or GOLD 2 to 4) because we were only interested in the heterogeneity of developing dementia between normal spirometry and PRISm. We performed a competing risk analysis for the influence of death treated as a competing risk using the method proposed by Fine and Gray.^36^ As a sensitivity analysis, we used the lower limit of normal (LLN) threshold of FEV_1_/FVC, where FEV_1_/FVC lower than LLN was used to define AFL.^30^^,^^37^ We also performed analyses restricted to non-users of bronchodilators, non-users of ICSs, participants with NT-proBNP <300 pg/mL, participants without kidney dysfunction, or participants without MCI at baseline and with additional adjustment for handgrip strength and total energy intake. In addition, we conducted analyses with an age-squared term to account for a potential nonlinear age effect on the outcomes.

All statistical analyses were performed with SAS statistical software, version 9.4 (SAS Institute Inc., Cary, NC, USA). Two-sided values of *P* < 0.05 were considered statistically significant in all analyses.

## RESULTS

### Prevalence and characteristics of each lung function category

The baseline characteristics of participants according to lung function category are shown in Table 1. Among 1,202 participants, the crude prevalences of PRISm, AFL GOLD 1, and AFL GOLD 2 to 4 were 11.4% (*n* = 137), 11.1% (*n* = 134), and 13.9% (*n* = 167), respectively. The group of participants with PRISm had significantly higher BMI and higher proportions of participants with BMI ≥25.0 kg/m^2^, hypertension, antihypertensive agents, dyslipidemia, lipid-modifying agents, diabetes mellitus, kidney dysfunction, history of stroke, and serum NT-proBNP ≥300 pg/mL than those with normal spirometry. The value of each spirometric parameter in the participants with PRISm was significantly lower than the corresponding value in participants with normal spirometry. The participants with AFL GOLD 1 and AFL GOLD 2 to 4 had significantly older age, lower eGFR, a higher proportion of men, and higher proportions of participants with smoking habits, bronchodilators, and ICSs than those with normal spirometry.

**Table 1.** Baseline characteristics of participants according to the lung function categories

	Lung function categories			

Characteristics	Normal spirometry	PRISm	AFL GOLD 1	AFL GOLD 2 to 4
	(*n* = 764)	(*n* = 137)	(*n* = 134)	(*n* = 167)
Age, years	72.9 (5.8)	73.1 (6.1)	75.0 (6.1)^a^	75.6 (6.6)^a^
Men, %	38.6	42.3	55.2^a^	63.5^a^
Education level of ≤9 years, %	35.3	43.1	31.3	37.1
BMI, kg/m^2^	23.2 (3.4)	24.6 (3.4)^a^	22.5 (2.5)	23.0 (3.4)
<18.5 kg/m^2^, %	7.5	3.6	4.5	8.4
≥25.0 kg/m^2^, %	26.4	45.3^a^	15.7^a^	25.7
Hypertension, %	67.1	79.6^a^	71.6	71.9
Systolic blood pressure, mm Hg	134 (19)	134 (17)	134 (16)	135 (18)
Diastolic blood pressure, mm Hg	77 (11)	75 (12)	76 (10)	75 (10)
Antihypertensive agents, %	51.6	67.2^a^	57.5	59.3
Dyslipidemia, %	58.4	74.5^a^	52.2	54.5
Serum total cholesterol, mmol/L	5.17 (0.88)	5.04 (1.05)	5.09 (0.95)	4.91 (0.95)^a^
Serum HDL cholesterol, mmol/L	1.68 (0.42)	1.60 (0.38)	1.65 (0.44)	1.55 (0.47)^a^
Lipid-modifying agents, %	33.4	50.4^a^	30.6	35.9
Diabetes mellitus, %	21.7	36.5^a^	26.9	26.9
Kidney dysfunction,%	5.9	12.4^a^	9.7	12.0^a^
eGFR, mL/min/1.73 m^2^	65.1 (11.8)	63.8 (13.8)	62.1 (11.0)^a^	61.5 (13.0)^a^
History of stroke, %	3.4	9.5^a^	5.2	5.4
Smoking status				
Current smoker, %	5.2	8.0	11.9^a^	16.8^a^
Former smoker, %	27.0	29.9	38.8^a^	47.9^a^
Never smoker, %	67.8	62.0	49.3^a^	35.3^a^
Pack-years^b^	31 (16–48)	41 (23–50)	35 (22–51)	40 (23–56)^a^
Current drinking, %	41.5	39.4	48.5	40.7
Regular exercise, %	19.9	22.6	26.9	17.4
Total energy intake, kcal/day	1,537 (342)	1,501 (326)	1,554 (299)	1,576 (354)
Handgrip strength, kg	27.5 (8.4)	26.9 (8.4)	28.9 (7.4)	29.0 (8.4)
Bronchodilators, %	1.7	2.2	8.2^a^	15.6^a^
Inhaled corticosteroids, %	0.5	2.2	3.7^a^	12.0^a^
Serum NT-proBNP ≥300 pg/mL, %	5.9	11.7^a^	8.2	15.1^a^
Serum NT-proBNP, pg/mL^c^	72 (44–121)	91 (41–164)	80 (51–145)	89 (52–172)^a^
Spirometric values				
FEV_1_, L	2.07 (0.50)	1.52 (0.34)^a^	1.99 (0.41)	1.44 (0.39)^a^
FEV_1_/FVC, %	76.7 (4.8)	75.1 (3.9)^a^	66.0 (3.2)^a^	61.4 (7.7)^a^
FEV_1_% predicted, %	98.0 (11.3)	72.5 (7.0)^a^	91.6 (8.8)^a^	65.1 (12.1)^a^
FVC, L	2.71 (0.65)	2.04 (0.47)^a^	3.02 (0.64)^a^	2.36 (0.61)^a^
FVC% predicted, %	99.3 (11.6)	75.1 (8.2)^a^	107.1 (10.5)^a^	81.9 (13.4)^a^

AFL, airflow limitation; BMI, body mass index; eGFR, estimated glomerular filtration rate; FEV_1_, forced expiratory volume in one second; FVC, forced vital capacity; GOLD, Global Initiative for Chronic Obstructive Lung Disease; HDL, high-density lipoprotein; NT-proBNP, N-terminal pro-B-type natriuretic peptide; PRISm, preserved ratio impaired spirometry.

Data are presented as the mean values (standard deviation) or percentages. Numbers of participants with missing data were as follows: 1 for education level, 2 for serum HDL cholesterol, 2 for pack-years, 4 for total energy intake, 1 for handgrip strength, and 3 for serum NT-proBNP and serum NT-proBNP ≥300 pg/mL.

^a^*P* < 0.05 vs normal spirometry.

^b^The values are shown as median values (interquartile range) of pack-years calculated in only current or former smokers.

^c^The values are shown as median values (interquartile range) of serum NT-proBNP.

### Lung function category and risk of development of dementia

**Figure 1.**
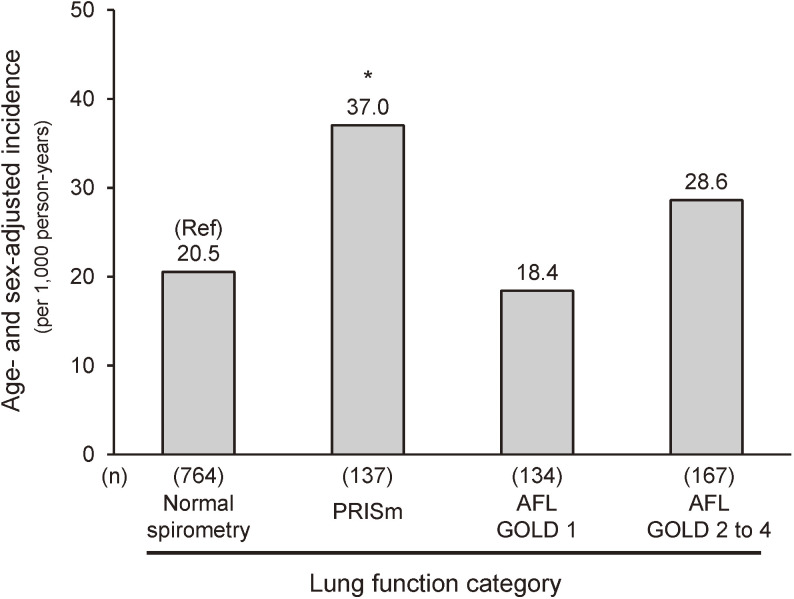
Age- and sex-adjusted incidence of dementia according to lung function category. AFL, airflow limitation; GOLD, Global Initiative for Chronic Obstructive Lung Disease; PRISm, preserved ratio impaired spirometry. ^*^P < 0.05 vs normal spirometry.

During the 5-year follow-up period, 122 (10.1%) participants developed dementia. Figure 1 showed the age- and sex-adjusted incidence rates of dementia according to the lung function category. The incidences of dementia per 1,000 person-years for normal spirometry, PRISm, AFL GOLD 1, and AFL GOLD 2 to 4 were 20.5, 37.0, 18.4, and 28.6, respectively. Table 2 shows the estimated HRs and 95% CIs for the development of dementia according to the lung function category. The age- and sex-adjusted HRs for developing dementia were significantly higher in the participants with PRISm compared to those with normal spirometry (model 1: HR 1.87; 95% CI, 1.12–3.12). This association remained significant after additional adjustment for potential confounding factors, such as education level, body mass index, systolic blood pressure, use of antihypertensive agents, serum total cholesterol, serum HDL cholesterol, lipid-modifying agents, diabetes, history of stroke, current smoking, pack-years, current drinking, and regular exercise (model 2: HR 2.04; 95% CI, 1.19–3.49). In contrast, the risk of dementia was not clearly elevated in the participants with AFL (GOLD 1 or GOLD 2 to 4) compared with those with normal spirometry. We also examined the association between each lung function category and development of dementia using the criterion of the LLN threshold for determining AFL. The results of these alternative analyses were similar to those of the main analysis (eTable 1). Moreover, similar associations were observed in the analyses with an additional adjustment for squared age entered into the models (eTable 2). We also performed sensitivity analyses among various subgroups of participants (ie, non-users of bronchodilators, non-users of ICSs, participants with serum NT-proBNP level <300 pg/mL, participants without kidney dysfunction, participants without MCI), and with additional adjustment for handgrip strength and total energy intake. The results of these analyses were also not substantially changed (eTable 3 and eTable 4). Moreover, the competing risk analyses using Fine and Gray models with a competing risk of death for dementia confirmed that the association between PRISm and dementia was not materially changed (eTable 5).

**Table 2.** Hazard ratios for the development of dementia according to lung function categories

Lung function categories	Number of events/ participants	Model 1		Model 2	
	
		Hazard ratio (95% CI)	*P* value	Hazard ratio (95% CI)	*P* value
Normal spirometry	66/764	1.00 (reference)		1.00 (reference)	
PRISm	19/137	1.87 (1.12–3.12)	0.02^a^	2.04 (1.19–3.49)	0.01^a^
AFL GOLD 1	13/134	0.86 (0.47–1.56)	0.61	0.91 (0.49–1.67)	0.75
AFL GOLD 2 to 4	24/167	1.25 (0.77–2.01)	0.37	1.39 (0.85–2.27)	0.19

AFL, airflow limitation; CI, confidence interval; GOLD, Global Initiative for Chronic Obstructive Lung Disease; PRISm, preserved ratio impaired spirometry.

Model 1: Adjusted for age and sex.

Model 2: Adjusted for age, sex, education level, body mass index, systolic blood pressure, use of antihypertensive agents, serum total cholesterol, serum high-density lipoprotein cholesterol, lipid-modifying agents, diabetes, history of stroke, current smoking, pack-years, current drinking, and regular exercise.

^a^*P* < 0.05 vs normal spirometry.

The subgroup analyses for various risk factors were performed among 901 participants with normal spirometry or PRISm (Table 3). There was significant heterogeneity in the association of PRISm with the risk of dementia only for alcohol intake: the association was stronger in the alcohol drinkers than in the non-drinkers (*P* for heterogeneity = 0.04). No evidence of heterogeneity was found in the association of PRISm with dementia risk among the risk-factor subgroups.

**Table 3.** Age- and sex-adjusted hazard ratios and their 95% confidence intervals for the development of dementia between normal spirometry and preserved ratio impaired spirometry in subgroups of the study population

Subgroups	Persons at risk	Number of events	Age- and sex-adjusted		*P* for heterogeneity
			HR (95% CI)	*P* value	
***Age***					
Age 65 to 74 years	585	25	1.35 (0.51–3.62)	0.54	0.57
Age ≥75 years	316	60	1.94 (1.05–3.56)	0.03^a^	
***Sex***					
Women	548	50	1.13 (0.50–2.52)	0.77	0.13
Men	353	35	2.86 (1.40–5.82)	0.004^a^	
***Education level***					
>9 years	572	43	1.80 (0.85–3.78)	0.12	0.88
≤9 years	328	42	1.90 (0.91–3.96)	0.09	
***BMI***					
<25.0 kg/m^2^	637	59	2.40 (1.19–4.86)	0.01^a^	0.40
≥25.0 kg/m^2^	264	26	1.53 (0.66–3.54)	0.32	
***Hypertension***					
No	279	10	1.12 (0.14–9.01)	0.91	0.67
Yes	622	75	1.70 (0.99–2.91)	0.054	
***Dyslipidemia***					
No	353	37	1.66 (0.68–4.04)	0.27	0.77
Yes	548	48	1.93 (1.01–3.69)	0.046^a^	
***Diabetes mellitus***					
No	685	60	1.62 (0.82–3.22)	0.17	0.59
Yes	216	25	2.55 (1.09–5.98)	0.03^a^	
***History of stroke***					
No	862	79	1.72 (0.99–2.99)	0.06	0.57
Yes	39	6	2.97 (0.52–16.99)	0.22	
***Smoking history***					
Never smoker	603	58	1.92 (0.98–3.74)	0.06	0.82
Current or former smoker	298	27	1.95 (0.84–4.52)	0.12	
***Alcohol intake***					
No	530	55	1.14 (0.55–2.35)	0.72	0.04
Yes	371	30	3.73 (1.72–8.09)	<0.001^a^	
***Regular exercise***					
No	718	68	1.96 (1.08–3.56)	0.03^a^	0.86
Yes	183	17	2.18 (0.74–6.44)	0.16	

BMI, body mass index; CI, confidence interval; HR, hazard ratios.

The participants with airflow limitation (*n* = 301) were excluded from the analyses.

^a^*P* < 0.05 vs normal spirometry.

### The severity of lung function and development of dementia

Finally, we investigated the association between each lung function parameter using spirometry, such as FEV_1_/FVC, FEV_1_% predicted, and FVC% predicted, and the risk for the development of dementia (Table 4). The participants in the lowest quartile of FEV_1_% predicted levels or those in the lowest quartile of FVC% predicted levels were at increased risk of dementia compared with those in the highest quartile after adjustment for the potential confounders. When estimating the risk of development of dementia per 1-standard deviation decrement in each lung function value, the HRs of the development of dementia increased significantly with decreases in the FEV_1_% predicted value or FVC% predicted value. There were no clear associations between the FEV_1_/FVC level and the risk for the development of dementia.

**Table 4.** Hazard ratios for the development of dementia according to each spirometric parameter

	Number of events/ participants	Model 1		*P* for trend	Model 2		*P* for trend
	
		Hazard ratio (95% CI)	*P* value		Hazard ratio (95% CI)	*P* value	
***FEV_1_/FVC level, %***							
Q4	26/300	1.00 (Reference)		0.98	1.00 (Reference)		0.65
Q3	28/302	1.15 (0.68–1.97)	0.60		1.27 (0.73–2.20)	0.40	
Q2	31/300	1.06 (0.63–1.79)	0.83		1.12 (0.66–1.89)	0.67	
Q1	37/300	1.04 (0.62–1.73)	0.88		1.18 (0.70–2.00)	0.53	
1-SD decrement		0.95 (0.80–1.14)	0.58		0.98 (0.82–1.18)	0.84	
***FEV_1_% predicted level, %***							
Q4	28/300	1.00 (Reference)		0.01	1.00 (Reference)		0.003
Q3	15/301	0.68 (0.36–1.27)	0.22		0.68 (0.36–1.29)	0.24	
Q2	36/301	1.40 (0.85–2.30)	0.18		1.51 (0.91–2.51)	0.11	
Q1	43/300	1.59 (0.98–2.57)	0.06		1.79 (1.09–2.94)	0.02^a^	
1-SD decrement		1.25 (1.07–1.47)	0.006		1.29 (1.09–1.52)	0.002	
***FVC% predicted level, %***							
Q4	23/300	1.00 (Reference)		0.002	1.00 (Reference)		0.001
Q3	26/301	1.32 (0.75–2.31)	0.34		1.24 (0.70–2.20)	0.45	
Q2	29/301	1.37 (0.79–2.37)	0.26		1.34 (0.77–2.35)	0.30	
Q1	44/300	2.21 (1.33–3.67)	0.002^a^		2.33 (1.39–3.93)	0.001^a^	
1-SD decrement		1.32 (1.13–1.56)	<0.001		1.35 (1.14–1.59)	<0.001	

CI, confidence interval; FEV_1_, forced expiratory volume in one second; FVC, forced vital capacity; SD, standard deviation.

Model 1: Adjusted for age and sex.

Model 2: Adjusted for age, sex, education level, body mass index, systolic blood pressure, use of antihypertensive agents, serum total cholesterol, serum high-density lipoprotein cholesterol, lipid-modifying agents, diabetes, history of stroke, current smoking, pack-years, current drinking, and regular exercise.

^a^*P* < 0.05 vs the reference (Q4).

## DISCUSSION

The current study demonstrated that PRISm was significantly associated with an increased risk of dementia in an older Japanese population. This association remained unchanged even after adjustment for confounding factors and several sensitivity analyses, with an alternative diagnostic criterion as the LLN threshold for the definition of AFL and excluding the participants with higher serum NT-proBNP or kidney dysfunction. Moreover, lower FEV_1_% predicted and FVC% predicted values, as indices of the severity of lung function, were associated with increased risks of the development of dementia. To the best of our knowledge, this is the first study addressing the risks of dementia in participants with PRISm in Japanese as a subgroup of the East Asian population.

Our findings were consistent with a previous report from the Rotterdam Study which found that PRISm was significantly associated with dementia.^4^ We also demonstrated a significant association between dementia and the severity of lung function in each of FVC% predicted and FEV_1_% predicted, but not in FEV_1_/FVC ratio. Consistent with our present results, several previous studies reported that FEV_1_% predicted and FVC% predicted levels were associated with dementia, but the FEV_1_/FVC ratio was not.^4^^,^^6^ These previous studies support our finding of an association between PRISm and the development of dementia, because PRISm is a subtype of lung function impairment with reduced FEV_1_ and FVC but preserved FEV_1_/FVC ratio. PRISm has been an ignored or overlooked subgroup of lung function impairment in previous studies, but these findings highlight the clinical importance of PRISm for identifying individuals at high risk for the development of dementia, and not only for those at risk of cardiovascular diseases and mortality.

The possible mechanisms underlying the association between PRISm and the development of dementia are unknown. One possible mechanism is that PRISm is an indicator of the accumulation of risk factors for dementia. Comorbidities of PRISm, including obesity, diabetes, and hypertension,^12^^–^^20^ are also common risk factors for dementia.^1^^,^^3^ Indeed, the present study showed that the participants with PRISm had higher proportions of these comorbidities. In addition, the decline in FEV_1_ (mainly caused by smoking) and the decline in FVC (factors that preserved the FEV_1_/FVC ratio) have also been reported to contribute to the higher risk for dementia.^4^^,^^6^ Lower FVC values have been caused by decreased thoracic compliance due to abdominal distention, decreased muscle strength and reduced total lung capacity via fluid shifts or cardiomegaly in heart failure or kidney dysfunction.^38^^–^^42^ These comorbidities and causes/risk factors with lower lung functions would be additively associated with the development of dementia (eFigure 2).^1^^,^^3^^,^^43^^,^^44^ However, the association between PRISm and the risk for developing dementia remained significant even after the multivariable adjustment for these comorbidities or in the sensitivity analyses excluding the participants with higher serum NT-proBNP (an indicator of heart failure) or kidney dysfunction. As another potential mechanism, PRISm has also been associated with a higher frequency of lacunar infarction,^45^ and many of the comorbidities in PRISm are consistent with risk factors for vascular dementia. These common risk factors for cerebrovascular disease and PRISm would be responsible for the potential mechanisms in the association between PRISm and the development of dementia. Other mechanisms, such as systemic inflammation, hypoxemia, and the duration of these comorbidities, might also have influenced the results. Further studies will be needed to investigate the mechanisms of PRISm and the development of dementia.

In the subgroup analysis, we found significant heterogeneity between PRISm and alcohol intake. The exact mechanism responsible for this heterogeneity in the association between subgroups of alcohol intake is unclear, and this finding may simply be due to chance. Nevertheless, it has been reported that excessive alcohol intake affects brain atrophy and cognitive decline^46^^,^^47^ as well as lung function decline,^48^ especially a decline in FEV_1_. Therefore, it is possible that alcohol intake enhances the brain damage and subsequent cognitive impairment in individuals with PRISm. Further studies in a population with drinking habits will be needed to clarify the association among alcohol consumption, PRISm and the development of dementia.

Several previous studies have examined the association between AFL and the development of dementia, but their conclusions have been inconsistent.^4^^–^^10^ In the present study, there were no clear associations between AFL (GOLD 1 or GOLD 2 to 4) and the risk for developing dementia. Unlike our participants with AFL, our participants with PRISm included many individuals with potentially reduced FVC in addition to FEV_1_, and the comorbidities associated with decreased FVC (eg, fluid retention due to heart failure or renal dysfunction, frailty, malnutrition, and interstitial lung disease) may be involved in the association between PRISm and dementia risk. Therefore, we conducted a sensitivity analysis excluding participants who were suspicious for heart failure and kidney dysfunction and with the additional adjustment for handgrip strength and total energy intakes as indicators of frailty and malnutrition, but the significant associations remained largely unchanged. Therefore, the influence of these comorbidities on the association between PRISm and dementia was likely to be modest. On the other hand, the possibility of residual confounding by interstitial lung disease could not be ruled out due to the lack of available data.

The strengths of the present study were its longitudinal population-based design, high participation rate in the baseline cognitive function survey (93.6%), and absence of loss to follow-up among study participants. Several limitations of the present study should also be noted. First, this study was based on spirometry without a bronchodilator, which could have led to misclassification of PRISm and AFL if participants with asthma was treated as having AFL. However, the association between PRISm and the risk of dementia remained significant even in the sensitivity analysis for non-users of ICSs or bronchodilators (eTable 3). Second, although prodromal dementia cases at baseline might have been included in the present study, a sensitivity analysis excluding participants with MCI did not alter any of the results (eTable 3). Third, residual confounding, such as that due to obstructive sleep apnea, pulmonary hypertension, or systemic inflammation, could have influenced the results. Fourth, participants excluded from the analysis due to missing data on physical measurements, laboratory measurements, or spirometry indices tended to be older and less educated than those included in the present study (data not shown). These selection biases may have affected the magnitude of the association between PRISm and AFL and the risk of developing dementia. However, we believe that they would not have substantially altered the association between PRISm and dementia. Finally, the present study was conducted in a single Japanese community, and therefore the generalizability of our findings to other populations with different backgrounds and lifestyles, such as non-Japanese populations or populations from other regions, is limited. Further research on the association between PRISm and dementia in other ethnic groups, or in other community, multi-community, or multinational studies is needed to improve the external validity and generalizability and to provide a more comprehensive understanding of the relationship between PRISm and dementia.

### Conclusion

In conclusion, the present study demonstrated that participants with PRISm had a significantly higher risk of dementia than those with normal spirometry. The result suggests that PRISm, a previously overlooked lung function category, is an important subtype of lung function impairment with a high risk of the development of dementia in a clinical setting. Further epidemiological studies in other ethnicities or races will be needed to elucidate the association between PRISm and the risk for developing of dementia.

## SUPPLEMENTARY MATERIAL

eFigure 1. Flowchart of participants

eFigure 2. Potential mechanisms underlying the association between PRISm and the development of dementia. FEV_1_, forced expiratory volume in one second; FVC, forced vital capacity; PRISm, preserved ratio impaired spirometry.

eTable 1. Hazard ratios for the development of dementia according to lung function categories with lower limit of normal threshold for airflow limitation

eTable 2. Hazard ratios for the development of dementia according to lung function categories: sensitivity analysis with additional adjustment for squared age

eTable 3. Hazard ratios for the development of dementia according to lung function categories: sensitivity analysis for several subgroups of participants

eTable 4. Hazard ratios for the development of dementia according to lung function categories: sensitivity analysis with additional adjustment for handgrip strength and total energy intake

eTable 5. Subdistribution hazard ratios for the development of dementia according to lung function categories using the Fine and Gray competing risk regression model with death as a competing risk
