# Supplementary material for: Risks of Dementia in a General Japanese Older Population With Preserved Ratio Impaired Spirometry: The Hisayama Study
Source: J Epidemiol. 2024 Jul 5;34(7):331–9. doi: 10.2188/jea.JE20230207 (PMC11167264; doi:10.2188/jea.JE20230207)
Supplement: Supplementary file 1 [file je-34-331-s001.pdf]

eFigure 1. Flowchart of participants

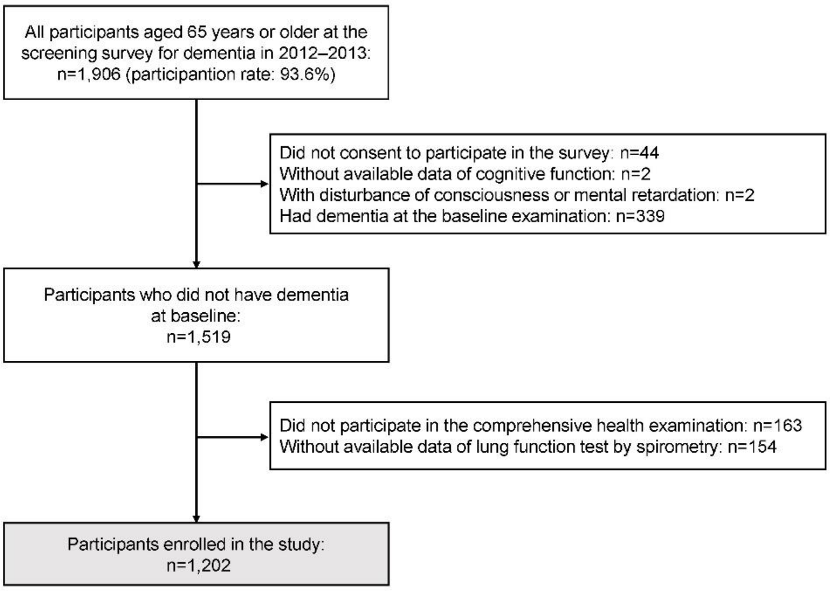

eFigure 2. Potential mechanisms underlying the association between PRISm and the development of dementia. FEV_1, forced expiratory volume in one second; FVC, forced vital capacity; PRISm, preserved ratio impaired spirometry.

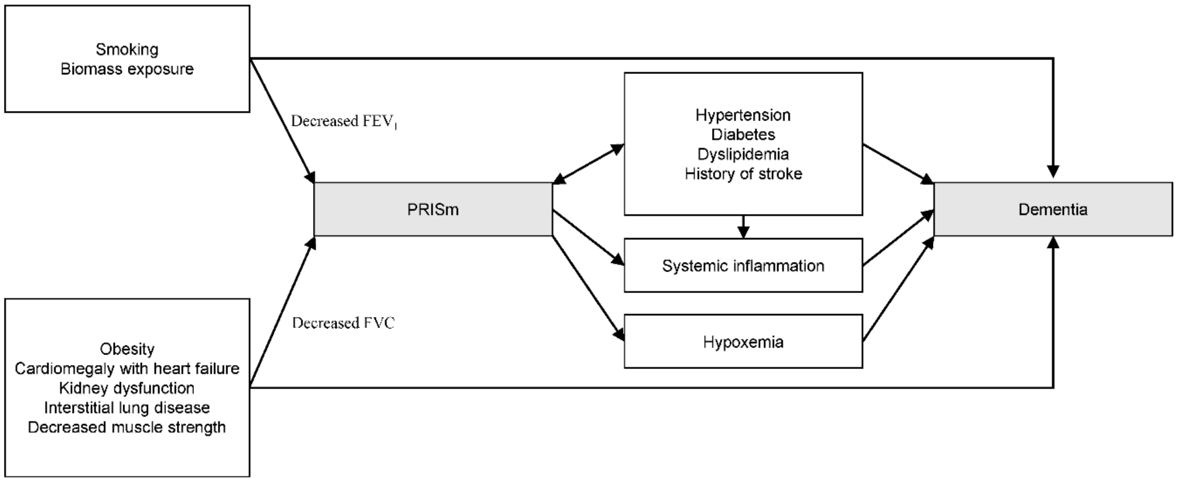

eTable 1. Hazard ratios for the development of dementia according to lung function categories with lower limit of normal threshold for airflow limitation

Lung function categories	Number of events /participants	Model 1		Model 2	
		Hazard ratio (95% CI)	*P* value	Hazard ratio (95% CI)	*P* value
Normal spirometry	73/816	1.00 (reference)		1.00 (reference)	
PRISm	28/183	1.64 (1.06–2.55)	0.03[a]	1.87 (1.18–2.97)	0.007[a]
AFL GOLD 1	6/82	0.80 (0.35–1.84)	0.60	0.88 (0.38–2.05)	0.77
AFL GOLD 2 to 4	15/121	1.26 (0.72–2.20)	0.42	1.32 (0.74–2.35)	0.35

AFL, airflow limitation; CI, confidence interval; GOLD, Global Initiative for Chronic Obstructive Lung Disease; PRISm, preserved ratio impaired spirometry.
Model 1: Adjusted for age and sex.
Model 2: Adjusted for age, sex, education level, body mass index, systolic blood pressure, use of antihypertensive agents, serum total cholesterol, serum high-density lipoprotein cholesterol, lipid-modifying agents, diabetes, history of stroke, current smoking, pack-years, current drinking, and regular exercise.
[a] $P<0.05$ vs. normal spirometry.

eTable 2. Hazard ratios for the development of dementia according to lung function categories: sensitivity analysis with additional adjustment for squared age

Lung function categories	Number of events /participants	Model 1		Model 2	
		Hazard ratio (95% CI)	*P* value	Hazard ratio (95% CI)	*P* value
Normal spirometry	66/764	1.00 (reference)		1.00 (reference)	
PRISm	19/137	1.83 (1.10–3.07)	0.02[a]	2.00 (1.16–3.42)	0.01[a]
AFL GOLD 1	13/134	0.82 (0.45–1.49)	0.52	0.85 (0.46–1.58)	0.61
AFL GOLD 2 to 4	24/167	1.17 (0.73–1.90)	0.51	1.31 (0.80–2.15)	0.28

AFL, airflow limitation; CI, confidence interval; GOLD, Global Initiative for Chronic Obstructive Lung Disease; PRISm, preserved ratio impaired spirometry.
Model 1: Adjusted for age, squared age and sex.
Model 2: Adjusted for age, squared age, sex, education level, body mass index, systolic blood pressure, use of antihypertensive agents, serum total cholesterol, serum high-density lipoprotein cholesterol, lipid-modifying agents, diabetes, history of stroke, current smoking, pack-years, current drinking, and regular exercise.
[a] $P<0.05$ vs. normal spirometry.

eTable 3. Hazard ratios for the development of dementia according to lung function categories: sensitivity analysis for several subgroups of participants

Lung function categories	Number of events/participants	Model 1		Model 2	
		Hazard ratio (95% CI)	*P* value	Hazard ratio (95% CI)	*P* value
Analyses for participants with non-use of bronchodilators					
Normal spirometry	66/751	1.00 (reference)		1.00 (reference)	
PRISm	19/134	1.87 (1.12–3.12)	0.02[a]	1.98 (1.15–3.39)	0.01[a]
AFL GOLD 1	12/123	0.83 (0.45–1.55)	0.56	0.86 (0.46–1.61)	0.63
AFL GOLD 2 to 4	23/141	1.36 (0.84–2.21)	0.21	1.47 (0.89–2.42)	0.13
Analysis for participants with non-use of inhaled corticosteroids					
Normal spirometry	66/760	1.00 (reference)		1.00 (reference)	
PRISm	19/134	1.90 (1.14–3.17)	0.01[a]	2.04 (1.19–3.50)	0.01[a]
AFL GOLD 1	12/129	0.82 (0.44–1.53)	0.54	0.87 (0.46–1.63)	0.65
AFL GOLD 2 to 4	23/147	1.35 (0.83–2.20)	0.23	1.49 (0.90–2.46)	0.12
Analysis for participants with serum NT-proBNP level <300 pg/mL					
Normal spirometry	59/719	1.00 (reference)		1.00 (reference)	
PRISm	16/121	1.88 (1.08–3.28)	0.03[a]	2.00 (1.10–3.61)	0.02[a]
AFL GOLD 1	10/123	0.83 (0.42–1.64)	0.60	0.85 (0.43–1.70)	0.65
AFL GOLD 2 to 4	21/142	1.31 (0.79–2.19)	0.30	1.43 (0.85–2.41)	0.18
Analysis for participants without eGFR <45 mL/min/1.73m²					
Normal spirometry	60/719	1.00 (reference)		1.00 (reference)	
PRISm	16/120	1.83 (1.05–3.19)	0.03[a]	1.95 (1.09–3.48)	0.02[a]

AFL GOLD 1	9/121	0.72 (0.35–1.45)	0.35	0.76 (0.37–1.57)	0.46
AFL GOLD 2 to 4	18/147	1.23 (0.72–2.10)	0.46	1.29 (0.74–2.23)	0.37
Analysis for participants without mild cognitive impairment at baseline					
Normal spirometry	43/682	1.00 (reference)		1.00 (reference)	
PRISm	15/125	2.19 (1.21–3.96)	0.009[a]	2.36 (1.27–4.41)	0.007[a]
AFL GOLD 1	11/117	1.12 (0.57–2.19)	0.74	1.26 (0.63–2.50)	0.51
AFL GOLD 2 to 4	16/137	1.37 (0.76–2.46)	0.30	1.66 (0.91–3.04)	0.10

AFL, airflow limitation; CI, confidence interval; eGFR, estimated glomerular filtration rate; GOLD, Global Initiative for Chronic Obstructive Lung Disease; NT-proBNP, N-terminal pro-B-type natriuretic peptide; PRISm, preserved ratio impaired spirometry.
Model 1: Adjusted for age and sex.
Model 2: Adjusted for age, sex, education level, body mass index, systolic blood pressure, use of antihypertensive agents, serum total cholesterol, serum high-density lipoprotein cholesterol, lipid-modifying agents, diabetes, history of stroke, current smoking, pack-years, current drinking, and regular exercise.
[a] $P<0.05$ vs. normal spirometry.

eTable 4. Hazard ratios for the development of dementia according to lung function categories: sensitivity analysis with additional adjustment for handgrip strength and total energy intake

Lung function categories	Number of events /participants	Model 1		Model 2	
		Hazard ratio (95% CI)	*P* value	Hazard ratio (95% CI)	*P* value
Normal spirometry	66/764	1.00 (reference)		1.00 (reference)	
PRISm	19/137	2.00 (1.16–3.43)	0.01[a]	2.01 (1.17–3.46)	0.01[a]
AFL GOLD 1	13/134	0.94 (0.51–1.73)	0.83	0.95 (0.51–1.75)	0.86
AFL GOLD 2 to 4	24/167	1.33 (0.81–2.19)	0.26	1.36 (0.82–2.24)	0.23

AFL, airflow limitation; CI, confidence interval; GOLD, Global Initiative for Chronic Obstructive Lung Disease; PRISm, preserved ratio impaired spirometry.

Model 1: Adjusted for age, sex education level, body mass index, systolic blood pressure, use of antihypertensive agents, serum total cholesterol, serum high-density lipoprotein cholesterol, lipid-modifying agents, diabetes, history of stroke, current smoking, pack-years, current drinking, regular exercise, and handgrip strength.

Model 2: Adjusted for model 1+ total energy intake.

[a] $P < 0.05$ vs. normal spirometry.

eTable 5. Subdistribution hazard ratios for the development of dementia according to lung function categories using the Fine and Gray competing risk regression model with death as a competing risk

Lung function categories	Number of events/death/ participants	Model 1		Model 2	
		SHR (95% CI)	*P* value	SHR (95% CI)	*P* value
Normal spirometry	66/37/764	1.00 (reference)		1.00 (reference)	
PRISm	19/12/137	1.75 (1.06–2.91)	0.03[a]	1.93 (1.12–3.34)	0.02[a]
AFL GOLD 1	13/9/134	0.86 (0.48–1.53)	0.60	0.91 (0.49–1.68)	0.76
AFL GOLD 2 to 4	24/20/167	1.22 (0.75–1.98)	0.43	1.36 (0.82–2.25)	0.24

AFL, airflow limitation; CI, confidence interval; GOLD, Global Initiative for Chronic Obstructive Lung Disease; PRISm, preserved ratio impaired spirometry; SHR, subdistribution hazard ratio.

Model 1: Adjusted for age and sex.

Model 2: Adjusted for age, sex, education level, body mass index, systolic blood pressure, use of antihypertensive agents, serum total cholesterol, serum high-density lipoprotein cholesterol, lipid-modifying agents, diabetes, history of stroke, current smoking, pack-years, current drinking, and regular exercise.

[a] $P<0.05$ vs. normal spirometry.
